# A Novel Microchip Flow Chamber (Total Thrombus Analysis System) to Assess Canine Hemostasis

**DOI:** 10.3389/fvets.2020.00307

**Published:** 2020-06-02

**Authors:** Tomoko Iwanaga, Naoki Miura, Benjamin M. Brainard, Marjory B. Brooks, Robert Goggs

**Affiliations:** ^1^Joint Faculty of Veterinary Medicine, Kagoshima University, Kagoshima, Japan; ^2^Department of Small Animal Medicine and Surgery, College of Veterinary Medicine, University of Georgia, Athens, GA, United States; ^3^Department of Population Medicine and Diagnostic Sciences, Cornell University College of Veterinary Medicine, Ithaca, NY, United States; ^4^Department of Clinical Sciences, Cornell University College of Veterinary Medicine, Ithaca, NY, United States

**Keywords:** canine, bleeding, platelets, hemophilia, von Willebrand's disease, Scott syndrome, flow chamber

## Abstract

Hemorrhagic diseases are common in dogs. Current coagulation assays do not model all aspects of *in vivo* hemostasis and may not predict bleeding risk. The Total-Thrombus Analysis System (T-TAS) is a novel hemostasis assay system in which whole blood flows through microfluidic channels at defined shear rates to provide qualitative and quantitative evaluation of platelet function (PL-chip) and coagulation function (AR-chip). The present study evaluated the T-TAS in dogs with hereditary bleeding disorders and with acquired hemorrhagic syndromes (Group 1), and healthy controls (Group 2). Hereditary defects included von Willebrand's disease (VWD; *n* = 4), hemophilia A (*n* = 2), and canine Scott syndrome (*n* = 2). Acquired hemorrhagic disorders included neoplastic hemoperitoneum (*n* = 2) and acute hemorrhagic diarrhea syndrome (*n* = 1). Citrate anticoagulated samples were collected from diseased dogs (Group 1, *n* = 11) and controls (Group 2, *n* = 11) for coagulation screening tests, fibrinogen analyses, D-dimer concentration, antithrombin activity, von Willebrand Factor antigen, PFA-100 closure time (PFA-CT), and thromboelastography (TEG). Citrate and hirudin anticoagulated samples were used for T-TAS analyses at two shear rates. Qualitative thrombus formation in each chip was recorded using the T-TAS video camera. Numeric parameters, derived from the instrument software, included occlusion start time (OST; time to 10 kPa), occlusion time (OT; time to 60 kPa (PL-chip) or 80 kPa (AR-chip)), and area under the pressure curve (AUC). Correlations between continuous variables were evaluated by Spearman's rank. Continuous variables were compared between groups by Student's *t*-test or the Mann-Whitney *U*-test. Alpha was set at 0.05. In combined analyses of all dogs, significant correlations were identified between T-TAS variables, between the PFA-CT and PL-chip parameters and between TEG variables and AR-chip parameters. The prothrombin time correlated with the AR-chip AUC at both shear rates. In Group 1 dogs, the AR-chip AUC at low shear was significantly reduced compared with Group 2 dogs. Aberrant thrombus formation was seen in video images recorded from dogs with VWD and hemophilia A. The T-TAS AR-chip analysis distinguished dogs with bleeding risk compared to healthy controls. Initial evaluations of the T-TAS suggest it may aid characterization of hemostasis in patients at-risk of bleeding and assist with delineating bleeding phenotypes.

## Introduction

Bleeding disorders are commonly encountered in veterinary medicine (1), and can be challenging to investigate and manage (2). Hemostasis *in vivo* occurs through assembly of coagulation factor complexes on the surfaces of activated platelets and tissue factor-bearing cells (3, 4). This cell-based localization of the hemostatic process enables sufficient thrombin to be generated to overcome the effects of dilution by blood flow and the presence of plasma and endothelial anticoagulants.

Although the cell-based model describes the mechanisms of hemostasis *in vivo*, it is difficult to apply to clinical diagnosis because of the complex interdependence of cellular and plasma factors. Bleeding disorders are typically categorized clinically as defects of platelet number and function (primary hemostasis) or defects of coagulation proteases (secondary hemostasis). Routine tests of hemostasis traditionally evaluate these components in isolation (5). For instance, primary hemostatic disorders are typically investigated by measuring platelet count, followed by testing the ability of platelets to become activated, to aggregate, to release the contents of their secretory granules and to express a procoagulant surface. Platelet function testing is often performed using isolated platelets, in non-clotting conditions and in the absence of blood flow. Secondary hemostatic disorders are typically investigated using plasma-based coagulation tests where the endpoint is the time to generate fibrin. Combined evaluation of the prothrombin time (PT), activated partial thromboplastin time (aPTT), and fibrinogen aids in localizing coagulation disorders to the contact pathway, tissue-factor pathway or the common pathway. Specific quantitative and functional factor assays are then used to further define single or combined factor deficiencies or identify the presence of factor inhibitors (5).

Patients with complex hemostatic defects may be difficult to diagnose and manage using these traditional laboratory tests. Test results are often poorly predictive of bleeding risk and do not reflect overall hemostatic balance. Standard tests also ignore the role of erythrocytes in clot formation and stability (6). The whole blood viscoelastic tests, including thromboelastography (TEG), thromboelastometry (ROTEM) and dynamic viscoelastic coagulometry (Sonoclot) were developed to address some of these shortcomings (7, 8), and may aid in predicting bleeding risk (9). Major limitations of the viscoelastic tests are their insensitivity to platelet function disorders (10, 11), and the static nature of the tests that ignores rheology, blood flow, and shear effects (12).

The Total Thrombus Analysis System (T-TAS) is a novel microfluidic system that measures whole blood flowing at defined, variable shear rates under conditions designed to assess platelet function or coagulation and fibrin clot formation (13). The T-TAS uses whole blood samples that flow through microfluidic chambers coated with collagen and tissue thromboplastin (atherome or AR-chip) or collagen only (platelet or PL-chip), to focus the evaluation on coagulation or platelet function, respectively (14–16). The system enables quantitative analysis of the timing, extent and rate of thrombus formation and is equipped with a video microscope for real-time imaging and qualitative analyses (17).

In people, the T-TAS system has been used extensively to assess the effects of antithrombotic drugs (14, 18), including oral direct Factor Xa inhibitors (19) and antiplatelet agents (20–22). Studies of patients with coronary artery disease treated with various antithrombotic therapies suggest the T-TAS can predict bleeding risk (23, 24). Consistent with its ability to detect impaired thrombus formation due to antiplatelet drug therapy, the T-TAS system is also sensitive to some inherited platelet function disorders. Patients with platelet storage pool disease (characterized by a reduction in the number or content of alpha granules or dense granules) have decreased thrombus formation in the PL-chip assay, at both low and high shear (17). In patients with von Willebrand's disease (VWD), the T-TAS appears to be sensitive to moderate to severe plasma von Willebrand factor (VWF) deficiency and the absence of large molecular weight VWF multimers (25). Samples from patients with severe type 1 VWD, defined as VWF antigen (VWF:Ag) below 10U/dL, and those with type 2 VWD (absence of large VWF multimers) failed to occlude either AR- or PL-chips but the occlusion times were normal in patients with milder type 1 VWD. In a study of type 1 VWD, patients with relatively prolonged T-TAS PL-chip T10 values had lower VWF levels (26). Moreover, PL-chip T10 values correlated with bleeding scores and bleeding severity, suggesting that T-TAS may aid prediction of bleeding risk among patients with type 1 VWD. Published data also suggest the T-TAS can identify hemostatic abnormalities due to coagulation factor deficiencies (27) and essential thrombocythemia (28).

To date, three studies have used the T-TAS to study canine samples. The first evaluated T-TAS reproducibility and established reference intervals in healthy dogs. The PL-chip assay parameters had coefficients of variation (CV) of 6.5–13.6%, while the AR-chip assay parameters had CV values of 1.6–10.0%. This study also assessed the effect of aspirin in 3 dogs and found that the PL-chip assay, but not the AR-chip assay, was affected by antiplatelet therapy (29). In two related experimental studies of atrial fibrillation induced by rapid atrial pacing, the T-TAS was used to evaluate the propensity of atrial thrombus development (30, 31). The time to initial clot formation (AR-chip T10) of blood collected from the right atrium and the occlusion time (OT) of both AR- and PL-chips were significantly shorter after 30 min of pacing compared to baseline. In contrast, no significant changes were observed in contemporaneous peripheral blood samples (31).

No studies to date have evaluated this system in canine patients with clinical bleeding disorders. The present study therefore aimed to perform T-TAS analyses in parallel with conventional hemostatic testing in dogs with hereditary and acquired bleeding disorders. It was hypothesized that T-TAS measures of platelet-mediated and coagulation-mediated thrombus formation correlate with relevant parameters derived from conventional plasma-based coagulation tests, platelet function analyses, and thromboelastography.

## Materials and Methods

### Animals

Two groups of dogs were recruited for the present study. Criteria for inclusion in Group 1 (dogs with acquired or hereditary bleeding disorders) included hereditary bleeding disorders such as VWD, diagnosed based on genotyping for the presence of a splice site mutation in the VWF gene (32) or deficiency of circulating VWF (VWF:Ag <50% of a canine pooled plasma standard) (33) and Hemophilia A, defined by severe deficiency of coagulation factor VIII (FVIII coagulant activity <10% of canine pooled plasma) (34, 35). Canine Scott syndrome was diagnosed based on abnormal platelet phosphatidylserine externalization and lack of microvesiculation (36). In addition, dogs with clinical bleeding caused by spontaneous hemoperitoneum or acute hemorrhagic diarrhea syndrome were included in this group. Spontaneous hemoperitoneum was defined as the presence of a hemorrhagic abdominal effusion (packed cell volume greater than 20%) in the absence of trauma, thrombocytopenia, recent antiplatelet agent therapy or known coagulopathy (37). The diagnosis of acute hemorrhagic diarrhea syndrome was based on the presence of acute onset bloody diarrhea in the absence of underlying disorders such as intestinal parasitism, hypoadrenocorticism, neoplasia or pancreatitis (38). Group 2 dogs (healthy controls) were eligible for inclusion if they had no history or evidence of recent or chronic medical conditions, had not received any medication, except for routine preventative healthcare, within the preceding 3 months, and had normal complete blood count and serum biochemistry results. Dogs weighing less than 5 kg were excluded to minimize the risk of additional blood sample collection.

### Blood Sampling and Routine Clinicopathologic Testing

Blood samples were collected by peripheral venipuncture into evacuated tubes using 21 g butterfly catheters (Surflo, Terumo, Somerset, NJ). A no-additive tube (BD Vacutainer, BD Biosciences, San Jose, CA) was drawn prior to collection of other samples. These tubes were discarded for dogs in Group 1 and used for serum chemistry testing for Group 2 dogs. For coagulation analyses, PFA-100 closure time (PFA-CT), and thromboelastography, 1.8 mL or 2.7 mL blood was collected into tubes containing 3.2% sodium citrate in a 1:9 ratio (Vacuette, Greiner Bio-One, Monroe, NC). For T-TAS analyses using AR-chips blood samples were collected into 3.2% citrate, while for T-TAS analyses using PL-chips blood samples were collected into dedicated sample tubes containing hirudin (Hirudin blood tubes, Diapharma, West Chester, OH). Samples for complete blood counts were collected last into K_2_-EDTA tubes (BD Vacutainer, BD Biosciences, San Jose, CA). Complete blood cell counts were performed on healthy controls using an ADVIA 2120 (Siemens, Washington, D.C.). Serum chemistry profiles were performed on healthy controls using a Cobas 501 (Roche Diagnostics, Indianapolis, IN). Blood cell counts and biochemical analyses were performed at the institution clinical pathology laboratory.

### Coagulation Testing and Factor Assays

Citrated plasma for clotting time tests and hemostatic protein analyses was prepared by centrifugation of whole blood at 10,000 × g for 10 min. The assays were performed at the Comparative Coagulation Laboratory at the Animal Health Diagnostic Center using automated and semi-automated coagulation instruments (STACompact and START4, Diagnostica Stago, Parsippany, NJ). A mechanical endpoint method and human coagulation reagents were used to measure clottable (Clauss) fibrinogen concentration (Fibrinogen, Diagnostica Stago), PT (Thromboplastin LI, Helena Diagnostics, Beaumont, TX), and aPTT (Dade Actin FS, Dade Behring, Newark, DE). Antithrombin (AT) activity was measured with a synthetic chromogenic substrate kit (Stachrom AT III, Diagnostica Stago) and D-dimer concentration was measured in a quantitative, turbidimetric immunoassay (HemosIL D-dimer, Instrumentation Laboratory, Lexington, MA). A pooled plasma prepared from healthy dogs (*n* = 20) was used as calibration standard for the fibrinogen and AT assays. The fibrinogen content of the plasma standard was determined by gravimetic method (39), and its AT activity was defined as 100%. The D-dimer assay was calibrated with a human D-dimer standard (HemosIL D-dimer calibrator, Instrumentation Laboratory, Bedford, MA). Plasma VWF:Ag was measured in an ELISA configured with monoclonal anti-canine VWF antibodies (40). Factor VIII coagulant activity (FVIII:C) was measured in a modified aPTT, using a human congenital Factor VIII deficient plasma (George King Biomedical, Overland Park, KS). The standard curves for VWF:Ag and FVIII:C were derived from dilutions of the canine standard which had an assigned value of 100% VWF:Ag and 100% FVIII:C.

### Platelet Function Analyses (PFA-100)

Platelet function under high-shear conditions was evaluated using the PFA-100 instrument (Siemens HealthCare Diagnostics, Deerfield, IL) according to the manufacturer's instructions. Cartridges containing collagen and ADP as platelet activators (COL/ADP) were used for this study. Citrated whole blood samples were gently mixed and then 800 μL was pipetted into the cartridge sample reservoirs for closure time measurement. Assays were performed in duplicate and the mean value used for subsequent statistical analyses.

### Thromboelastography (TEG)

Rotational viscoelastic testing was performed with a computerized instrument (TEG 5000 Hemostasis Analyzer, Haemoscope, Niles, IL) using recalcified, nonactivated-citrated blood (citrate-native) and recalcified-citrated blood activated with recombinant human tissue factor (TF), as previously described (41, 42). Assays were conducted in accordance with the PROVETS guidelines (43, 44). In brief, reaction cups warmed to 37°C were loaded with 20 μL of 280 mM CaCl_2_ and either 340 μL of citrated blood or 340 μL of citrated blood containing a TF-phospholipid reagent (Dade Innovin, Siemens Healthcare Diagnostics, Tarrytown, NY) diluted 1:50,000 in the final (360 μL) reaction mixture (42). The TEG analyses on nonactivated and TF-activated blood were performed simultaneously in 2 channels for 60-minute run times with compilation of the following TEG parameters: reaction time (R), clotting time (K), angle (α), maximal amplitude (MA), global clot strength (G), and time to maximal rate of thrombus generation (TMRTG) (41).

### Thrombin Generation (TG)

Banked aliquots of citrate plasma stored at −80°C (for up to 10 months) were used for thrombin generation assays by the calibrated automated thrombogram method. Thrombin generation was measured in an integrated spectrofluorimeter/analytic software instrument (Thrombinoscope, Diagnostica Stago) using the manufacturer's TG reagents (PPP-low, Thrombin calibrator, FLUCa), as previously described (45, 46). Briefly, the assay measures thrombin formation over time in tissue-factor activated, recalcified citrated plasma based on cleavage of a fluorogenic thrombin substrate. Thrombin generation in the test plasma is calculated by comparing fluorescence levels in the tissue factor activated-sample with a paired plasma reaction containing a thrombin calibrator. Coagulation and calibration reactions were performed in triplicate, with 80 μL PPP (diluted 1:2 in buffer solution) reacted with either 20 μL tissue factor reagent (containing phospholipids and 1 pM human recombinant tissue factor) or 20 μL thrombin calibrator. Thrombin generation was monitored for 60 min at 10 s intervals in the coagulation and calibrator wells after the addition of 20 μL of a solution containing calcium (CaCl_2_) in HEPES buffer (pH 7.35), and the fluorescent substrate. Reactions were performed in round bottom, high binding polystyrene microtiter plates (Immulon 2HB, Thermo Scientific, Waltham, MA). Aliquots of canine pooled plasma were included on each test plate as a reagent control. The Thrombinoscope software generates qualitative tracings (thrombograms) and numeric parameters, including the following parameters compiled for statistical analyses: (1) Lag time, defined as the time from assay initiation to the beginning of thrombin generation. (2) Peak, defined as the maximum quantity of thrombin generated during the reaction (nM). (3) Endogenous thrombin potential (ETP), defined by the area under the TG curve and representing the total amount of thrombin formed over 60 min.

### Evaluation of Thrombus Formation Under Flow Conditions

*Ex vivo* thrombus formation was analyzed using a T-TAS instrument (Fujimori Kogyo, Tokyo, Japan) using two types of analysis chips; the PL-chip (channel width 40 μm × depth 40 μm) (containing 25 capillary channels coated with type-I collagen) and the AR-chip (width 300 μm, depth 60 μm, length 15 mm) (consisting of a single capillary channel coated with collagen and thromboplastin). The PL-chip analyzes platelet-rich thrombus formation under two shear rates (1500 s^−1^, and 2000 s^−1^) and the AR-chip analyzes fibrin-rich thrombus formation under two different shear rates (240 s^−1^ and 600 s^−1^). For PL-chip analysis, 320 μL hirudin-anticoagulated whole blood is pipetted into the sample reservoir, then perfused through the chip at 37°C by a pneumatic pump driving a column of mineral oil. In this assay, platelet activation and thrombus formation are initiated by shear and interactions between platelets and the collagen coating on the capillary channels. For AR-chip analysis, 480 μL citrate-anticoagulated whole blood is mixed with 20 μL of 0.3M calcium solution containing 1.25 mg/mL corn trypsin inhibitor (Fujimori Kogyo, Tokyo, Japan) and immediately pipetted into the reservoir. The recalcified blood is then perfused at 37°C through the AR-chip. During perfusion of blood through the capillary, PLs and the extrinsic coagulation pathway are simultaneously activated through exposure to collagen and tissue thromboplastin. In the AR-chip assay, the effluent blood from the chip is mixed with 25 mM EDTA (pH 10.5) solution to prevent occlusion. The process of thrombus formation in both chips is monitored by a pressure sensor located between the pump and the blood sample reservoir that detects flow pressure changes over time. As thrombus formation proceeds on its coated surface, the capillary is gradually occluded, thereby increasing the flow pressure. The following parameters were derived by the instrument's software from T-TAS tracings: (1) OST (occlusion start time or time to reach 10 kPa), defined as the time in minutes for the flow pressure to increase from baseline to 10 kPa due to partial occlusion of microcapillaries. This parameter defines the onset of thrombus formation. (2) OT (occlusion time), defined as the time for the flow pressure to reach 60 kPa (PL-chip) or 80 kPa (AR-chip). This parameter represents the time for complete occlusion of the capillary by thrombus. (3) T10–60 (PL-chip) and T10–80 (AR-chip), defined as the time interval between T10 and OT. This parameter represents the rate of thrombus growth. (4) AUC (area under the curve), defined as the area under the flow pressure curve from baseline to 10 min (PL-chip) or from baseline to 30 min (AR-chip). This parameter quantifies the amount of thrombus formation during the early part of the reaction if the pressure required to generate an occlusion time is not achieved.

### Statistical Analysis

Prior to test selection, data were assessed for normality using the D'Agostino Pearson test and descriptive statistics calculated as appropriate. Comparisons of continuous variables between groups were performed using Student's *t*-tests or the Mann Whitney *U*-test. Associations between continuous variables were evaluated by calculation of Spearman's rank correlation coefficient. Alpha was set at 0.05 and all analyses were conducted using commercial software (JMP 12.2, SAS Institute Inc. and Prism 8.3, GraphPad, La Jolla, CA).

## Results

### Animals

All dogs were enrolled at Cornell University with informed client consent under local IACUC approval. Eleven dogs were enrolled in each group. The bleeding disorder dogs (Group 1) consisted of 8 dogs with hereditary bleeding disorders: 2 Dobermans heterozygous for a VWF mutation with normal VWF:Ag and 1 Doberman deficient in VWF:Ag (VWD type 1), 1 Shetland sheepdog lacking detectable VWF (VWD type 3), 2 mixed breed dogs with Hemophilia A, and 2 dogs with Scott syndrome (*n* = 1 German shepherd, *n* = 1 Shepherd/Malinois mix). The type 1 VWD dogs had no clinical histories of abnormal bleeding. The dogs with hemophilia A, type 3 VWD, and the Scott syndrome German shepherd had all experienced one or more episodes of abnormal bleeding, but had been clinically stable with no active bleeding for at least 1 month before blood collection for the study. The Shepherd/Malinois dog with Scott syndrome had acute, severe epistaxis at the time of study entry. The 3 dogs with acquired defects had active hemorrhage, including 2 mixed breed dogs with neoplastic hemoperitoneum (hemangiosarcoma (*n* = 1), hepatocellular carcinoma and hemangiosarcoma (*n* = 1) and a Boston terrier with acute hemorrhagic diarrhea. The healthy control group dogs (Group 2) included 6 mixed breed dogs, 2 Labrador retrievers, and 1 each of 3 other pure breeds. All Group 2 dogs were deemed clinically healthy based on complete blood counts, serum biochemistry, and routine coagulation function testing.

### Coagulation Testing, VWF:Ag, PFA-100

**Figure 1:**
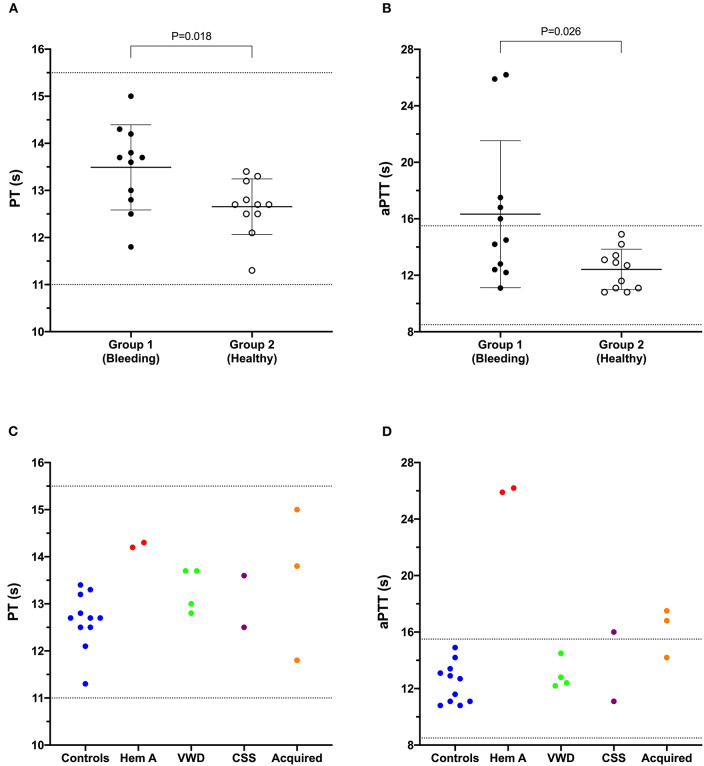
Dot plots comparing the prothrombin time (PT) **(A)** and activated partial thromboplastin time (aPTT) **(B)** values of Group 1 (bleeding risk) dogs with those of Group 2 (healthy control) dogs. Values for PT and aPTT in Group 1 were significantly longer than those in Group 2 (Student's *t*-test). Coagulation times from individual dogs are also plotted according to health status or disease process **(C,D)**. CSS, canine Scott syndrome; Hem A, hemophilia A; VWD, von Willebrand's disease.

Results of coagulation tests, VWF:Ag, and PFA100 closure times for Group 1 and Group 2 dogs are displayed in Table 1. As expected, the two dogs with hemophilia A had prolonged aPTT (Figure 1) and the two VWF-deficient dogs had maximally prolonged PFA-100 closure times beyond 300 s (Table 1). The dog with Scott syndrome and severe epistaxis had slight (0.5 s) prolongation of the aPTT, with marked elevation in fibrinogen and VWF:Ag. Two dogs with hemoperitoneum had marked increases in D-dimer and variably prolonged aPTT, and one had moderate increase in fibrinogen. A single dog in Group 2 had a D-dimer value that was mildly increased compared to the reference interval, but this dog was judged healthy based on physical examination and normal values for all other hemostasis tests (Table 1).

**Table 1:** Summary of the results of routine coagulation testing for the two groups of dogs.

**Gp**	**Diagnosis**	**Breed**	**Sex**	**Active bleeding** **Yes/No**	**aPTT (s) (8.5-15.5)**	**PT (s)** **(11.0-15.5)**	**Fg (mg/dL)** **(150-490)**	**AT (%) (65–145)**	**DD (ng/mL)** **(0-575)**	**VWF:AG (%)** **(70-180)**	**PFA-CT (s)** **(50–120)**
1	Type 1 VWD carrier (genetic test)	Doberman	F	No	12.8	12.8	286	123	217	109	77
	Type 1 VWD carrier (genetic test)	Doberman	M	No	12.4	13.7	272	118	312	113	100
	Type 1 VWD (VWF deficient)	Doberman	M	No	12.2	13.7	381	118	419	20	>300
	Type 3 VWD (VWF absent)	Shetland sheepdog	FS	No	14.5	13	368	101	413	0	>300
	Hemophilia A	GSD	M	No	26.2	14.2	287	107	83	146	77
	Hemophilia A	GSD	M	No	25.9	14.3	242	111	6	125	73
	Scott syndrome	Mix (GSD x Malinois)	M	Yes	16	13.6	1,434	108	63	387	73
	Scott syndrome	GSD	MC	No	11.1	12.5	332	109	191	177	56
	Hemoabdomen, Hemangiosarcoma	Mix	MC	Yes	16.8	11.8	676	80	4,511	245	81
	Hemoabdomen, Hepatocellular carcinoma Hemangiosarcoma	Mix	M	Yes	17.5	15	271	110	6,051	108	187
	Acute Hemorrhagic Diarrhea Syndrome	Boston terrier	FS	Yes	14.2	13.8	460	75	395	137	47
2	Healthy control	Boxer	FS	No	12.9	12.8	379	93	272	106	99
	Healthy control	Mix	F	No	11.1	12.7	346	103	86	136	87
	Healthy control	Mix	FS	No	14.2	12.5	291	116	92	245	66
	Healthy control	Labrador retriever	FS	No	13.1	12.7	226	105	351	154	62
	Healthy control	Irish wolfhound	FS	No	10.8	12.5	264	109	130	147	57
	Healthy control	Mix	FS	No	10.8	12.1	379	105	459	78	69
	Healthy control	Beagle	MC	No	14.9	11.3	290	125	182	100	92
	Healthy control	Mix	FS	No	11.6	13.2	229	127	270	94	66
	Healthy control	Mix (Mastiff x)	MC	No	13.4	13.3	263	115	788	133	60
	Healthy control	Mix (Labrador x)	FS	No	12.7	13.4	319	115	456	178	50
	Healthy control	Labrador retriever	FS	No	11.1	12.7	368	105	375	102	67

*Values highlighted in red text were increased above the reference interval. Values highlighted in blue were decreased below the reference interval*.

*aPTT, activated partial thromboplastin time; AT, antithrombin activity; DD, D-dimer; Fg, fibrinogen; Gp, Group; GSD, German shepherd dogs; PFA-CT, Platelet function analyzer-100 closure time; PT, prothrombin time; VWD, von Willebrand's disease; VWF:Ag, von Willebrand factor antigen*.

### Thrombelastography and Thrombin Generation

**Figure 2:**
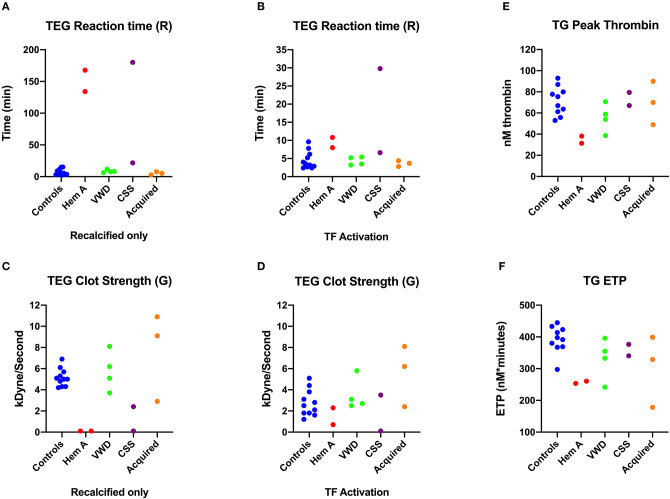
Scatterplots comparing key thromboelastography (TEG) and thrombin generation (TG) parameters among dogs in Group 1. **(A)** Reaction times (R-time) from the citrate recalcified (native) TEG assay. **(B)** Elastic shear modulus or clot strength values **(F)** from the citrate recalcified (native) TEG assay. **(C)** Reaction times from the tissue factor (TF) activated TEG assay. **(D)** Peak thrombin from the thrombin generation (TG) assay. **(E)** Endogenous thrombin potential (ETP) derived from the TG assay. CSS, canine Scott syndrome; Hem A, hemophilia A; VWD, von Willebrand's disease.

Overall, the recalcified only TEG reactions revealed more profound abnormalities in Group 1 dogs than the tissue-factor activated reactions (Figures 2A–C). Both dogs with hemophilia A, and the Scott syndrome dog with acute, severe hemorrhage, had essentially no bonding between the TEG cup and pin in the non-activated reactions, denoted by unmeasurable G (clot strength parameter) and marked prolongation of R (clot initiation parameter). This failure of clot formation in tissue factor reactions was only consistent for the Scott syndrome dog (Figures 2B,C). The dogs with acquired bleeding disorders had a wide range of TEG abnormalities. Although both dogs with neoplastic hemoperitoneum had prolonged aPTT, one had a high G value and the other a low G value, denoting increased or decreased clot strength relative to values for the controls. Parameters of thrombin generation also differed for these two dogs (Figures 2D,E). The dog with low clot strength also demonstrated relatively low values of peak thrombin formation and thrombin generating potential, suggesting that impaired fibrin formation was due to a failure of coagulation and inadequate fibrinogen cleavage. Two dogs with hemophilia A and one VWF deficient dog also demonstrated low thrombin generation, below that of controls (Figures 2D,E).

### T-TAS

**Figure 3:**
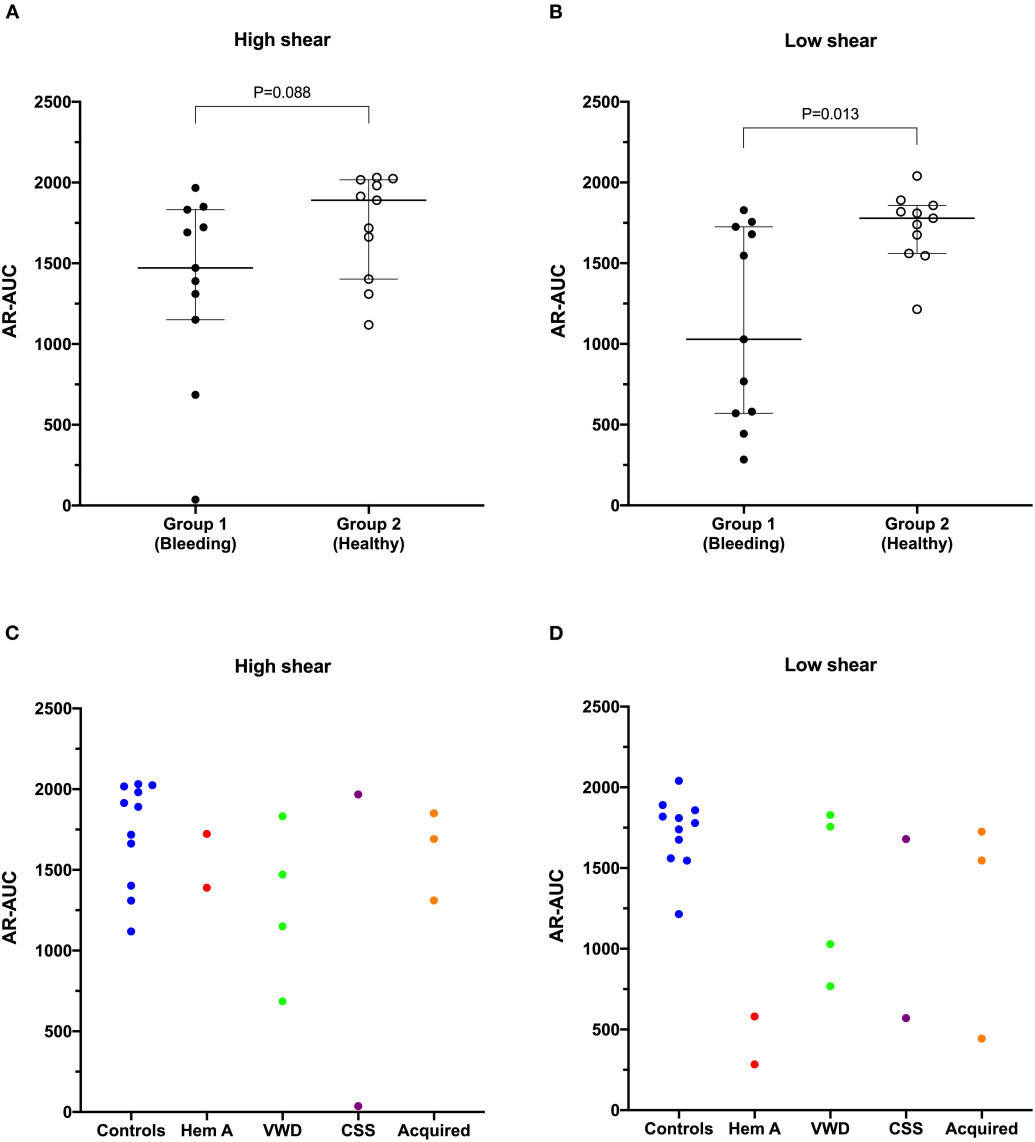
The area under the curve value derived from the AR-chip T-TAS assay was not significantly reduced in dogs in Group 1 compared to Group 2 at high shear **(A)**. However, under low shear conditions **(B)**, the area under the curve parameter from the AR-chip assay was significantly reduced in dogs at-risk of bleeding, compared to healthy controls (Mann-Whitney *U*-test). This suggests the low-shear assay may be more sensitive to the types of bleeding disorders present in the dogs in the study population than is the high-shear assay. Area under the curve values from individual dogs are also plotted according to health status or disease process **(C,D)**. AUC, area the under the curve; CSS, canine Scott syndrome; Hem A, hemophilia A; VWD, von Willebrand's disease.

**Figure 4:**
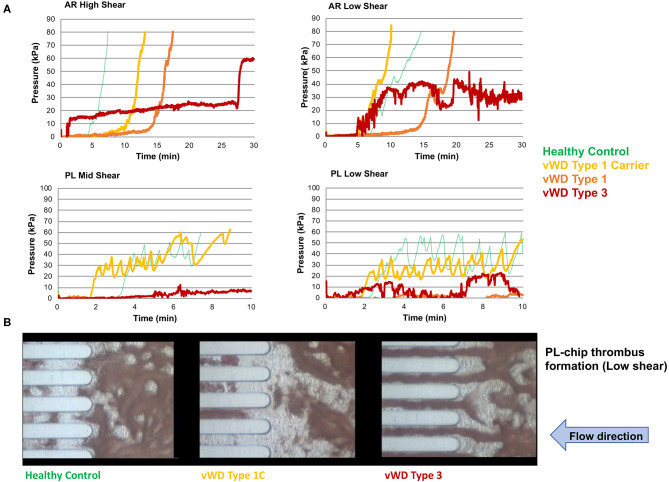
The T-TAS assay profiles and visualizations of thrombus formation in dogs with von Willebrand's disease (Type 1 carrier (normal VWF:Ag), Type 1 (low VWF:Ag), Type 3 (absent VWF:Ag)). **(A)** Pressure-time graphs from AR-chip and PL-chip assays of a healthy control and 3 dogs with VWD. In the AR-chip assays thrombus formation in dogs with type I VWD is delayed (graphs are right-shifted) particularly at high shear, and is incomplete in the dog with type 3 VWD at both high and low shear. The PL-chip profile from the type I VWD carrier dog was similar to that of the healthy control. In the PL-chip assay, the occlusion pressure failed to increase in VWF-deficient dogs (type 1 and type 3 VWD). **(B)** Representative images of thrombus formation within PL-chip microfluidic channels. Blood samples collected from healthy control dogs, a heterozygous carrier for a VWF mutation having normal VWF:Ag, and a dog lacking VWF protein (type 3 VWD) were flowed (from right to left) through the collagen coated microfluidic channels. Stable thrombi formed in samples from the control dog and VWD carrier resulting in occlusion of the microfluidic channels. In contrast, no thrombi formed within the PL-chip microchannels in the sample from the dog with type 3 VWD.

**Figure 5:**
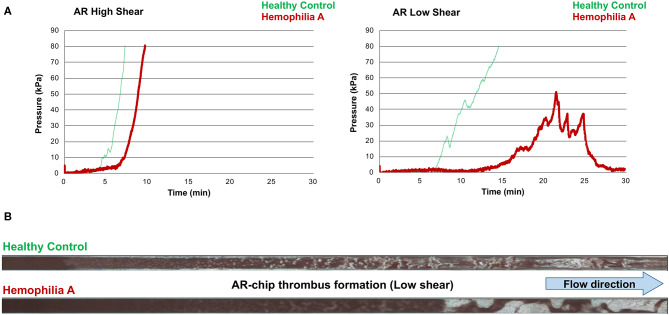
The T-TAS assay profiles and visualizations of thrombus formation in a dog with hemophilia A. **(A)** At high shear, the AR-chip occlusion profile was normal, while at low shear occlusion was delayed and the profile never reached the endpoint occlusion pressure. **(B)** Representative images of the single AR-chip flow channel with blood samples from a healthy control and a dog with hemophilia A flowed through the channel following activation by collagen and thromboplastin. The channel itself appears dark and the formed thrombi are white. Blood flow was from left to right. In these overview images of the whole AR-chip microchannel the thrombus build-up in the healthy control occurs early and causes complete occlusion. In contrast, in the dog with hemophilia A, thrombus formation can be seen only further down the flow channel and never became occlusive. This lack of occlusion occurred due to a residual flow path within the thrombus that developed due to ongoing blood flow through an unstable and incomplete blood clot.

Values for the AR-chip AUC at low shear were significantly lower for the Group 1 dogs vs. Group 2 controls (*P* = 0.018) (Figure 3). In dogs with VWD (Type 1 and Type 3) (Figure 4A), the T-TAS video images (Figure 4B) suggested that platelet crosslinking was diminished or abnormal. In samples from dogs with Type 1 and Type 3 VWD, thrombus did not readily form perpendicular to the blood flow direction. Thus, in dogs with Type 1 and Type 3 VWD accumulating platelet thrombi rarely occluded the microfluidic channels. In contrast, in dogs with Type 1 and Type 3 VWD platelet thrombi did form parallel to the blood flow direction, resulting in accumulation of platelets in front of and adjacent to the microfluidic channel dividers (Videos 1, 2). Healthy control dogs formed platelet thrombi parallel and perpendicular to the blood flow direction and therefore readily generated thrombi that occluded the microfluidic channels (Figure 4B). Profiles generated using the AR-chip at a high flow rate from a dog with hemophilia A showed thrombus formation at a normal rate and with normal morphology. In contrast, at low shear, thrombus formation in the dog with hemophilia A was abnormal and caused limited, transient increases in pressure within the flow channel (Figure 5). In one dog with hemoperitoneum and TEG tracings consistent with hypocoagulability (low G values), the AR-AUC at low shear was lower than the mean AR-AUC in healthy dogs (443.5 vs. 1721.2), while the AR-AUC at high shear was closer to the mean AR-AUC healthy dogs (1310.4 vs. 1733, respectively). This dog had a normal PT, but mild prolongation of the aPTT (17 s, with a high normal reference range of 15.5 s). Results from the PL-chip analysis in this dog were similar to the mean AUC of healthy dogs at both shear levels (medium: 220.9 vs. 340 and low: 150.8 vs. 275.8, respectively). Of interest, this dog, diagnosed with hepatocellular carcinoma, had a PFA-CT of 187 s, indicating some decreased platelet function that may have contributed to the low G of the TEG analysis, but which was not reflected in the PL-chip results. The other dog with hemoperitoneum, despite a mild prolongation of aPTT, had AR- and PL-AUC that were similar to the mean values from the healthy dogs, and had an increased recalcified TEG G value, indicating robust clot strength. A Boston terrier with hemorrhagic diarrhea also had a discordant result between the low shear PL-AUC (61.2 compared to a mean of 275.8 in healthy dogs), and a PFA of 47 s, representing a normal aperture occlusion time.

### Correlations Between Assays

**Figure 6:**
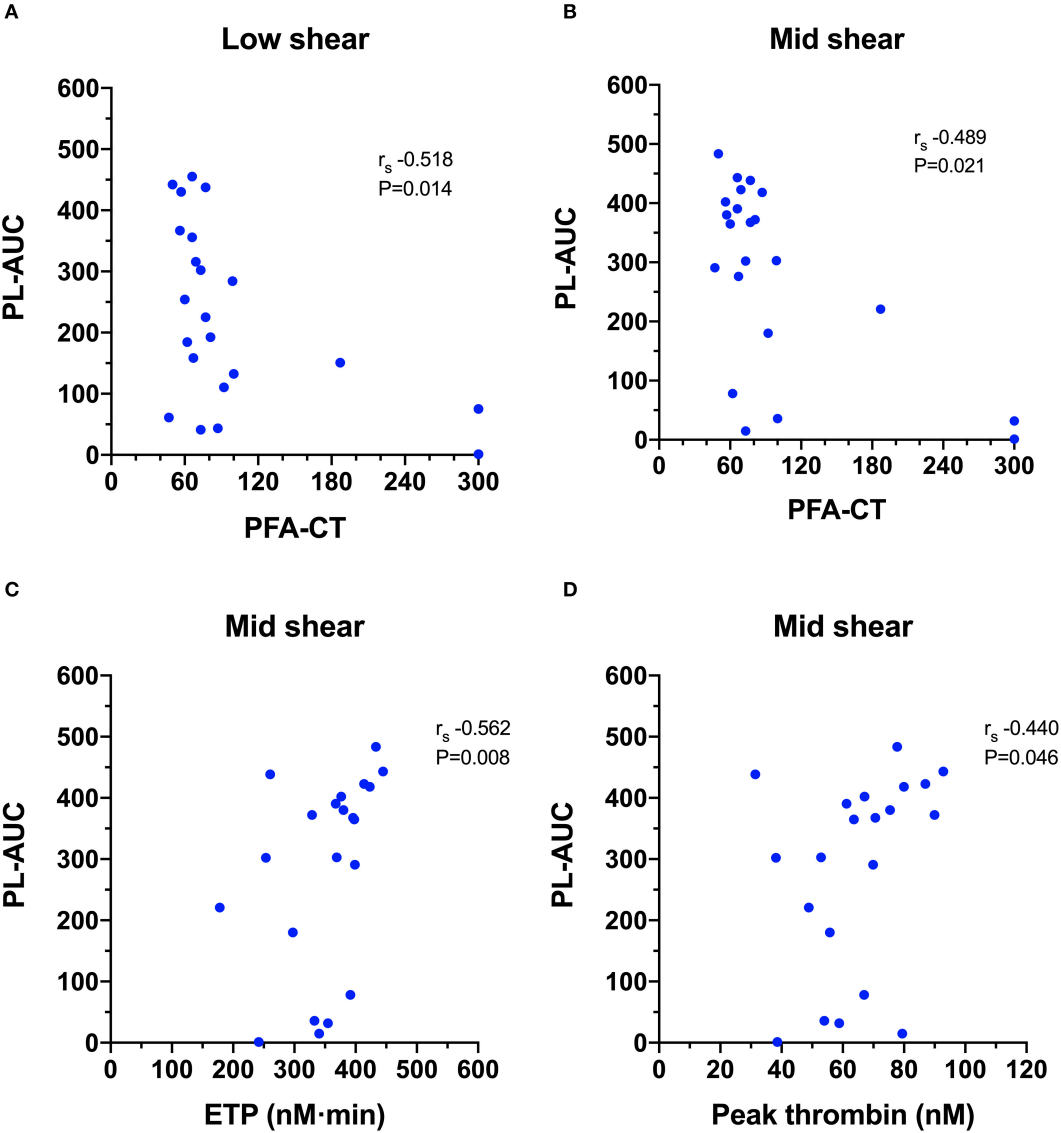
Scatterplots of T-TAS parameters derived from platelet chip (PL-chip) assays against those derived from routine coagulation tests. **(A,B)** The closure time of the platelet function analyzer (PFA-CT) was negatively correlated with the PL-chip area under the curve (PL-AUC) at both low and mid-shear suggesting that long closure times are associated with limited platelet thrombus formation under flow conditions. **(C,D)** The endogenous thrombin potential (ETP) and the peak thrombin generation from the thrombin generation assay were both positively correlated with the area under the curve values from the PL-chip at medium shear (PLM-AUC) suggesting that thrombin generation contributes to thrombus formation in the T-TAS assay under these conditions.

Calculation of Spearman's rank correlation coefficients between the T-TAS parameters and those generated from the other hemostasis testing identified several statistically significant and biologically plausible associations (Video 2). For the PL-chip (Figure 6), the PFA-CT negatively correlated with the PL-AUC at both low (r_s_ −0.518, *P* = 0.014) and mid shear rates (r_s_ −0.489, *P* = 0.021) and both assay platforms demonstrated failure of thrombus formation in VWF-deficient samples. There were two significant correlations between TG and PL-chip parameters. The PL-AUC at the mid shear rate positively correlated with the ETP (r_s_ 0.562, *P* = 0.008) and with peak thrombin generation (r_s_ 0.440, *P* = 0.046).

**Figure 7:**
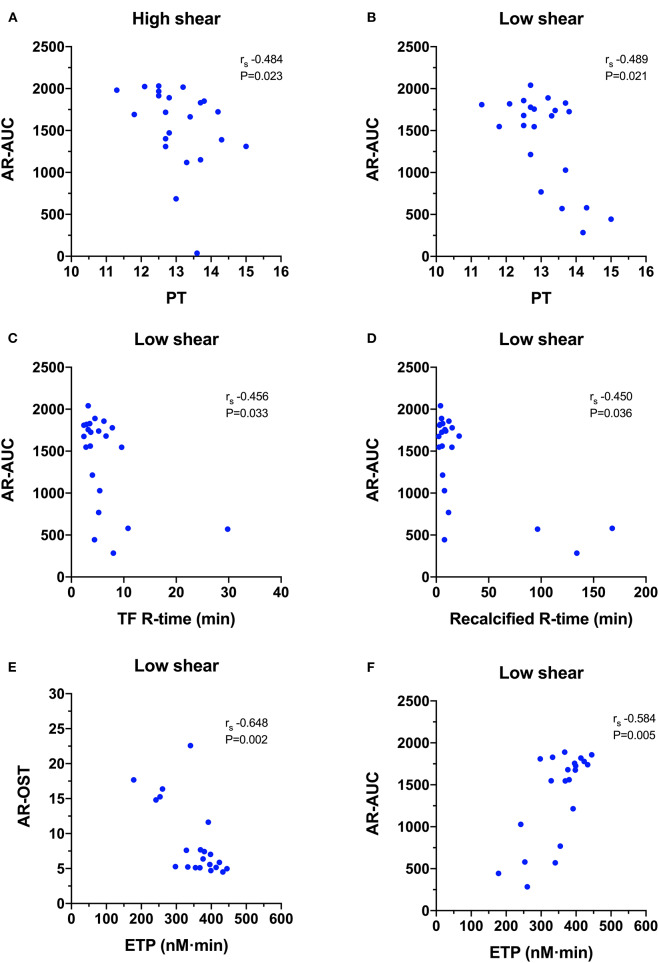
Scatterplots of T-TAS parameters derived atherome chip (AR-chip) assays against those derived from routine coagulation tests. **(A,B)** The area under the curve (AR-AUC) was negatively correlated with the prothrombin time (PT) at both high and low shear suggesting that long clotting times are associated with reduced extent of thrombus formation under flow conditions. **(C,D)** The AR-AUC parameter also negatively correlated with the reaction time from the tissue factor and recalcification thromboelastography assays. **(E,F)** The endogenous thrombin potential (ETG) derived from the thrombin generation assay was negatively correlated with the occlusion start time, and positively correlated with the area under the curve suggesting that the rate and the extent of thrombus formation in the AR-chip assay are related to thrombin generation.

For the AR-chip (Figure 7), the PT negatively correlated with the AR-AUC at both the high (r_s_ −0.484, *P* = 0.023) and the low shear (r_s_−0.489, *P* = 0.021) rates. There were several significant negative correlations with TEG parameters. The AR-AUC at low shear rate was significantly negatively correlated with the reaction time (R) for the TF activated assay (r_s_−0.456, *P* = 0.033) and for the recalcified only assay (r_s_−0.450, *P* = 0.036). There were three significant correlations between TG and AR-chip parameters. The AR-chip OST at the low shear rate significantly correlated with the ETP parameter (r_s_−0.648, *P* = 0.002) and the AR-AUC at the low shear rate significantly correlated with the ETP (r_s_ 0.584, *P* = 0.005) and with peak thrombin generation (r_s_ 0.469, *P* = 0.032).

## Discussion

The T-TAS is a flow chamber system that uses whole blood microchip-based flow assays to model *in vivo* hemostasis. The T-TAS quantitatively analyzes thrombus formation and also enables qualitative assessment of the thrombus formation under variable shear conditions. Data in people suggests the system can accurately assess primary hemostasis and predicts bleeding during percutaneous coronary interventions and arthroplasty. Additionally, the T-TAS can detect the therapeutic effects of dual antiplatelet therapy and of oral anticoagulants (22). In people, the system augments established plasma and whole blood assays of hemostasis. To date, studies in dogs are limited but the system is the first commercial assay that enables evaluation of primary and secondary hemostasis under flow conditions. Data provided by the present pilot study are preliminary, but suggest that the system may enable assessment of various hemostatic abnormalities in dogs.

In the present study, the T-TAS system revealed aberrant thrombus formation in dogs with defined, hereditary defects of primary and secondary hemostasis. Von Willebrand factor plays a critical role in supporting platelet adhesion to collagen; a property enhanced by VWF conformational changes under flow conditions. As described for human VWD patients, data presented here suggest the PL-chip assay may be sensitive to VWF deficiency in dogs. In addition to numeric parameters, the T-TAS video-microscopy feature generated dynamic images that suggest moderate to severe VWF deficiency impairs platelet thrombus stability. The AR-chip assay includes a thromboplastin coating that triggers coagulation, thus complementing examination of platelet function in the PL-chip. Samples from dogs with hemophilia A demonstrated a delay in clot initiation and weak, non-occlusive clot formation in the AR-chip low shear assay, thus confirming its sensitivity to detect procoagulant factor deficiency in this species. Our preliminary study of dogs with specific defects suggests that combined examination of the T-TAS system assays will provide insights into the mechanisms of aberrant thrombus formation in patients with more complex hemostatic disorders.

Analyses of combined results from all dogs revealed correlations between T-TAS parameters and the various traditional tests of the relevant pathway. The PFA-100 instrument is designed to assess platelet adhesion and aggregate formation under high shear conditions. The inverse association between the PL-chip AUC values and PFA-100 closure time indicates that the unstable platelet thrombi visualized in the T-TAS represent the same phenomenon underlying failure of membrane occlusion and prolongation of PFA-100 closure time. The markedly prolonged closure times and reduced PL-AUC for the two dogs with VWF deficiency are consistent with this critical need for VWF to support platelet adhesion under flow conditions. Flow chamber assays have been instrumental in investigations of the pathogenesis of VWD and of the interactions between VWF and platelet surface receptors (47–50). We also found a positive correlation between the thrombin generation parameters and the AUC for the PL-chip. Although the hirudin anticoagulant used for PL-chip samples blocks thrombin's ability to form fibrin in the flow chamber, some thrombin-mediated augmentation of platelet aggregation may occur. Stable, occlusive thrombi may form more rapidly, therefore, in samples with higher thrombin generating capacity.

The AR-chip assay initiates coagulation protein complex assembly and activation through contact with thromboplastin under flow conditions. Thus, the AR-chip assay is tuned to identify defects in the onset, propagation, and ultimate stability of fibrin thrombi. Assays focused on one or more of these processes, such as the prothrombin time and TEG, are therefore likely to demonstrate similar results. Accordingly, we found negative correlations between the AR-chip AUC parameter and clotting time in the PT and the clot reaction time (R-time) in the thromboelastography system. These correlations suggest that delays in forming the initial fibrin strands, representing the assay endpoints of the PT and R-time, are associated with reductions in occlusive thrombus formation under flow conditions. The cleavage of fibrinogen to yield fibrin requires generation of a thrombin burst (51) and correspondingly the AR-chip occlusion start time, and area under the curve parameters were correlated with the endogenous thrombin potential (52). These associations support the T-TAS system's ability to provide further insight into the influence of fluid phase procoagulant protein reactions on platelet and fibrin thrombus formation under flow.

Overall, the strength of the correlations identified between T-TAS parameters and conventional coagulation tests was weak to moderate. Hemostasis in whole blood under flow conditions at variable shear is complex and involves myriad interactions between platelets, plasma proteins and the flow chamber surfaces. In contrast, conventional hemostasis assays isolate specific aspects of the coagulation process to focus on detecting dysfunction in one component. It is therefore reasonable that the correlations between simplified conventional tests of one aspect of coagulation and the complex multidimensional whole blood flow assays are at best moderate. The whole blood flow assay may offer advantages for analysis of complex hemostatic disorders or the detection of disorders such as Scott syndrome where the defect exists in the interaction between platelets and the coagulation system. Additional studies will be necessary to confirm this supposition.

Previous studies in dogs have used flow chamber assays to evaluate platelet interactions with extracellular matrix components (53) and to assess the influence of bacterial infection, lipopolysaccharide and hydroxyethyl starch on platelet function (54). Flow chamber systems have also been used to study human patients with hemophilia, VWD and hereditary platelet function disorders including May-Hegglin anomaly, gray platelet syndrome, and Glanzmann thrombasthenia (55), all of which are disorders of relevance to canine medicine. The T-TAS system has been used to evaluate healthy dogs and to assess the effects of antiplatelet agents in people (56) and in dogs (29). In our current study of dogs with bleeding disorders, we found significant differences between healthy and diseased dogs in the AUC parameter only under low shear conditions, suggesting that limiting shear-induced platelet activation may enhance assay sensitivity (57). By plotting the individual disease processes out, it can also be seen that several of the dogs with VWD had reduced AUC values under high shear conditions (Figure 3C). This might reflect the shear-dependence of the interactions between wild-type VWF and the platelet receptors GPIb and GPIIb-IIIa (58, 59).

As a pilot project, a limitation of the study includes the heterogeneity of the hemostatic defects evaluated. While a breadth of testing was performed in every dog, the total number of dogs was small. Most canine hereditary bleeding disorders are rare, but the present study included dogs with a specific defect of fibrin formation, i.e., hemophilia A, and a specific platelet function defect i.e., VWD. The abnormalities noted in the T-TAS system assays recapitulated the underlying mechanism of hemostatic failure for each of these disorders. The platelet defect of Scott syndrome does not impair platelet adhesion or aggregation, and does not influence fibrin formation initiated by an excess of tissue thromboplastin. The traditional and T-TAS assays that monitor these processes showed no abnormalities in the Scott syndrome dog without active hemorrhage. In contrast, the Scott syndrome dog with severe, acute blood loss and epistaxis demonstrated delayed and weak clot formation in the TEG reactions, with corresponding low AUC value in the AR-chip assay. This finding suggests that aberrant *ex vivo* thrombus formation in the assay systems was reflective of *in vivo* hemostatic status. The T-TAS assay has good reproducibility in healthy dogs (29), but the inter-assay variability in disease states is not known and our study did not include serial sampling to evaluate consistency over time, or to evaluate response to therapy. Similarly, it is not known if all of the dogs with the bleeding disorders studied are truly representative of their condition. This is particularly the case with dogs that have acquired bleeding disorders such as those dogs with hemangiosarcoma since the bleeding disorders associated with cancer can be varied and complex (60–62). Two of the healthy control dogs in the present study each had one abnormal coagulation value (increased D-dimer or increased VWF:Ag). These dogs had no signs of disease and hence high values were considered to likely represent outliers in the population or transient increases without clinical relevance. High VWF:Ag was also found two dogs with active hemorrhage: one dog with Scott syndrome and severe epistaxis and one dog with hemangiosarcoma and hemoabdomen. High circulating levels of VWF, associated with an acute phase response, are seen in patients with endothelial injury and inflammatory syndromes (63). Additionally, it is possible that high plasma VWF in the dog with hemangiosarcoma was a result of tumor expression (64), while in the dog with acute hemorrhage due to Scott syndrome the increased VWF:Ag might have represented ADH and epinephrine stimulated release from endothelial cells in response to severe blood loss (65).

In dogs with clinical bleeding, the T-TAS results mirrored those from traditional coagulation testing, with some discrepancies, primarily between the PL-chip and the PFA results. In one case, a prolonged PFA-CT, indicating platelet hypofunction, was not reflected in the PL-chip AUC and in another the opposite occurred. Notably, the PL-chip was tested at low and moderate shear, (1500 s^−1^, and 2000 s^−1^) and not the high shear generated by the PFA (5000 s^−1^) (66). The combination of both testing modalities may thus be useful to diagnose complex platelet function abnormalities, for instance, as larger VWF molecules may become more important for platelet adhesion at higher shear rates. Another dog had the opposite scenario, with a rapid PFA-CT but lower than expected PL-AUC. This dog had experienced hypovolemic shock and was also experiencing gastrointestinal hemorrhage from acute hemorrhagic diarrhea syndrome. It is possible that this dog's platelets were activated by the high shear of the PFA, but were in fact hypofunctional at the lower PL-chip shear rates (the moderate shear PL-AUC was similar to healthy dogs).

The potential impact of red blood cell mass and platelet count on the T-TAS assay was not investigated by the present study. It is known that hematocrit and platelet count are important for TEG assays (67) and available data suggest that while platelet count does correlate with some AR-chip parameters the effect of variation in platelet count is limited (13). However, until more is known about the relationship between hematocrit and platelet count and T-TAS parameters in dogs, it might be prudent for future studies utilizing the T-TAS to adopt recommendations developed for TEG assays (44). Specifically, routine reporting of platelet count, hematocrit, and fibrinogen concentration in addition to the T-TAS results would be reasonable.

In summary, the T-TAS assay system detected and characterized primary and secondary hemostatic disorders in dogs. The numeric parameters derived from the T-TAS assays correlated with relevant parameters of other hemostatic tests of similar processes. The T-TAS video-microscopy capabilities also offer novel, qualitative information that complements traditional tests. Use of the two separate T-TAS assays at different shear rates provides the opportunity for detailed analyses of platelet function disorders and coagulation defects and to incorporate the influence of blood flow into the hemostatic process. Our preliminary results in a limited number of dogs with bleeding disorders suggest that future studies of the T-TAS system are warranted. Its unique combination of quantitative and qualitative assessment of thrombus formation will provide mechanistic insights into the pathophysiology of canine hemorrhagic disorders and may prove useful for diagnostic testing, prediction of bleeding severity, and gauging response to therapy.

## Data Availability Statement

All datasets generated for this study are included in the article/Supplementary Material.

## Ethics Statement

The animal study was reviewed and approved by Cornell University IACUC (Protocol numbers 2014-0052 and 2014-0053). Written informed consent was obtained from the owners for the participation of their animals in this study.

## Author Contributions

TI designed and conducted experiments, analyzed data and co-wrote the manuscript. NM designed experiments, analyzed data and co-wrote the manuscript. BB and MB designed and conducted experiments, analyzed data and edited the manuscript. RG designed experiments, analyzed data and wrote the manuscript.

## Conflict of Interest

The authors declare that the research was conducted in the absence of any commercial or financial relationships that could be construed as a potential conflict of interest.

## Supplementary Material

Video 1 Representative time-lapse videos of thrombus formation in the platelet-chip (PL-chip) assay in samples from a healthy control dog, two dogs with genetic defects associated with VWD (VWD Type 1c) and two dogs with clinical von Willebrand's disease (Type 1 and Type 3). Blood flow was horizontally from right to left. Blood flow was at low shear (1500s^−1^). Videos are 16× normal speed.

Video 2 Representative time-lapse videos of thrombus formation in the platelet-chip (PL-chip) assay in samples from a healthy control dog, two dogs with genetic defects associated with VWD (VWD Type 1c) and two dogs with clinical von Willebrand's disease (VWD Type 1 and Type 3). Blood flow was horizontally from right to left. Blood flow was at medium shear (2000s^−1^). Videos are 16× normal speed.

## Abbreviations

α-angle: clot formation angle
aPTT: activated partial thromboplastin time
AR-chip: atherome chip
AT: antithrombin activity
AUC: area under the curve
CV: coefficient of variation
Collagen / ADP: COL/ADP
ETP: endogenous thrombin potential
G: global clot strength
HEPES: 2-[4-(2-hydroxyethyl)piperazin-1-yl]ethanesulfonic acid
K-time: clotting time
MA: maximal amplitude
OST: occlusion start time
OT: occlusion time
PFA: platelet function analyzer
PFA-CT: PFA-100 closure time
PL-chip: platelet chip
PPP: platelet poor plasma
PROVETS: Partnership on Rotational ViscoElastic Test Standardization
PT: prothrombin time
R-time: reaction time
ROTEM: thromboelastometry
r_s_: Spearman's rank correlation coefficient
TEG: thromboelastography
TG: thrombin generation
TMRTG: time to maximal rate of thrombus generation
T-TAS: Total-Thrombus Analysis System
VWD: von Willebrand's disease
VWF:Ag: von Willebrand Factor antigen.
